# The Effects of Mechanical Loading on Resonant Response of a Conformal Load-Bearing Antenna System

**DOI:** 10.3390/s24196206

**Published:** 2024-09-25

**Authors:** Shouxun Lu, Kelvin J. Nicholson, Joel Patniotis, John Wang, Wing Kong Chiu

**Affiliations:** 1 Department of Mechanical & Aerospace Engineering, Monash University, Clayton, VIC 3800, Australia; wing.kong.chiu@monash.edu; 2 Defence Science and Technology Group, Aerospace Division, Fishermans Bend, VIC 3207, Australia; kelvin.nicholson@defence.gov.au (K.J.N.); joel.patniotis@defence.gov.au (J.P.); john.wang@defence.gov.au (J.W.)

**Keywords:** conformal load-bearing antenna system (CLAS), resonance frequency, relative permittivity, glass fibre-reinforced polymer (GFRP), fatigue response, mechanical testing

## Abstract

Glass fibre-reinforced polymer (GFRP) is a suitable substrate material for constructing a Conformal Load-Bearing Antenna Structure (CLAS). The relative permittivity of the CLAS substrate, which determines its resonant frequency, is affected by damage sustained by GFRP. This paper investigates the effects of damage (induced by mechanically loading the substrate) on the resonant response of the CLAS. Decoupling the antenna from the substrate was essential to evaluate the CLAS’s true response to the induced damage. This paper details a systematic investigation examining how the frequency response of a “pristine” antenna and a surface-mounted antenna respond to a substrate subjected to quasi-statically induced mechanical damage and cyclic fatigue loading. The results demonstrate that the resonant frequency of the CLAS varies as a function of the substrate’s mechanical damage. The prepared CLAS is tolerant to a certain degree of mechanical loading and related damage with its resonant frequency remaining within an acceptable bandwidth.

## 1. Introduction

**Figure 1:**
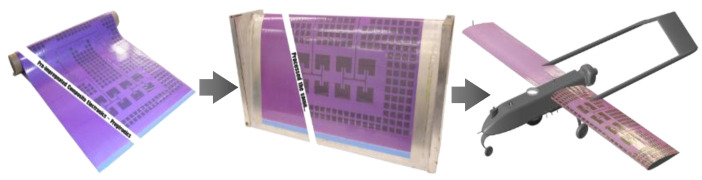
CLAS concept. Prepreg composite material with embedded electromagnetic traces can be cured in aerospace composite structures [4].

In the modern aerospace industry, the design and manufacturing process of antennas for large aerostructures is typically handled separately, with antennas mounted onto the exterior of the airframe. The antennas that protrude into the airflow create drag and exhibit less environmental damage tolerance during flight. This leads to a continuous drive towards multifunctional structures that can bear loads while providing communication and sensing capabilities. This trend is particularly evident in the development of CLASs, which integrate radiofrequency devices directly into the composite skins of airframes, as shown in Figure 1. Embedding radiofrequency devices into composite skins during manufacturing allows for strategic positioning along the exterior of a component, thereby freeing up internal space [1,2,3]. In addition, this integration offers the potential to radically enhance electromagnetic performance by distributing an array of elements across the entire aircraft. Moreover, with a CLAS embedded into the aerostructure, fuel efficiency is increased by reducing drag, and the system demonstrates significantly greater resilience to environmental damage compared to conventional antenna structures [2].

**Figure 2:**
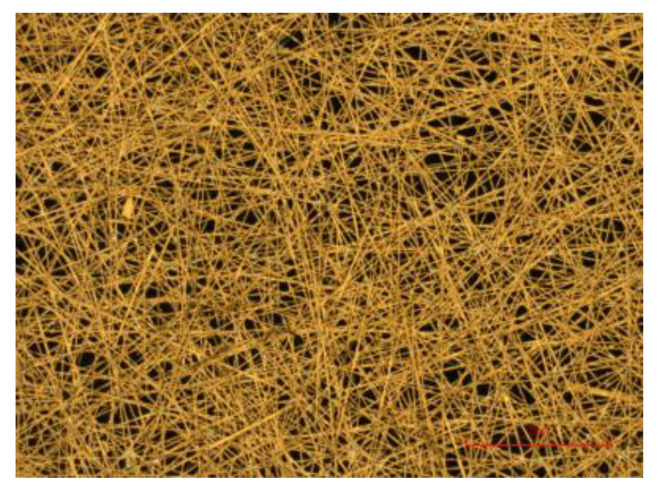
Close-up of copper-coated veil [4].

A thin copper sheet is the material of choice for microstrip antenna construction. However, this type of solid metal foil demonstrates significant degradation under mechanical loading [5]. Carbon-based non-woven fibres have the potential to replace copper in various applications, particularly in microstrip antennas [6,7,8]. Surface veils, consisting of randomly oriented, short-chopped carbon fibres, can be plated with highly conductive metals such as copper to achieve the desired electromagnetic properties, as shown in Figure 2 [9]. Additionally, due to their high-strength carbon core, these veil materials exhibit minimal degradation in conductivity performance under mechanical loading. This characteristic offers a distinct advantage over copper foil, which can lead to crack growth and debonding from the composite [10,11], and polymer core fibres, which tend to become open-circuit under high loading conditions [9]. This antenna material is favourable since it can be laser-cut and easily consolidated with traditional composite structures due to its high porosity [8,12].

Regarding the substrate of CLASs, You and Hwang proposed integrating a microstrip antenna into a composite sandwich structure. They used glass/epoxy as a face sheet material without adversely affecting the antenna efficiency. They noted that experimental results met the requirements for improved gain and wide bandwidth [13].

Due to the integration of radiofrequency devices into the aircraft structure, a CLAS will experience various types of loading under in-service conditions, such as wind, vibration, and impact, which induce structural deformation. Damage to the integrated antenna structure can affect not only the structural performance but also have varying impacts on their electromagnetic performance. It is, therefore, necessary to understand the effects of the loading experienced by the structure on the system’s functions to ensure the satisfactory performance of the radiofrequency system. Hence, we need to understand the effects of mechanical loading on the CLAS’s electromagnetic characteristics to ensure functionality.

Our study in [4] examines the mechanical and electromagnetic behaviour of load-bearing patch antennas under various mechanical loading conditions, such as tensile, biaxial, and twisting loads, through numerical simulations. The research highlights the sensitivity of the patch antenna’s resonant behaviour to mechanical deformations. Structural deformations, especially in the Y direction under biaxial loading, significantly alter the resonant frequency and reduce the quality factor, potentially degrading communication performance and leading to connectivity loss. Additionally, the study highlights that disbonds between the antenna and its substrate (a common issue arising from manufacturing defects or operational stresses) amplify these effects, with larger disbonds causing more pronounced shifts in resonant frequency.

Lu et al. [14] examine the effects of fatigue on the resonant frequency performance and quality factor of a CLAS. Experimental data reveal that a CLAS can maintain its performance within the operational band for up to 350k constant amplitude cycles with a peak strain of 4000 με applied at the edge of the antenna patch, with the resonant frequency remaining stable. However, performance begins to degrade significantly beyond this point, as evidenced by resonant frequency shifts and quality factor reductions, potentially leading to communication failures. Further investigation into cantilever loading on fatigued specimens shows that specimens without seeded defects remain within operational bands when subjected to bending tension, even after significant fatigue cycling. Conversely, specimens with seeded defects exhibit marked instability under static loading conditions. This instability leads to substantial performance degradation, rendering these antennas unusable after approximately 400k fatigue cycles. The primary cause of this instability is the accentuation of fatigue damage under static compressive loads, highlighting the critical impact of manufacturing defects on the long-term reliability of a CLAS.

These papers underscore the importance of meticulous quality control in the fabrication of a CLAS, highlighting that even minor defects can significantly impact performance under operational loads. Additionally, these findings emphasise the importance of testing and validation for this type of multifunctional composite structure. Understanding the effects of various loading conditions on the performance of a CLAS is crucial. This understanding is essential for ensuring a CLAS’s long-term reliability and durability during extended periods of in-air service.

The work presented in this paper seeks to understand the impact of mechanical loading conditions, such as quasi-static tension and cyclic loading for localised fatigue damage, on the electromagnetic properties of a CLAS. This paper introduces an approach to decouple the antenna from the substrate to systematically examine the response of a “pristine” antenna on a substrate subjected to accumulated quasi-static and cyclically induced mechanical damage. Subsequent experiments are conducted with surface-mounted antennas to compare results. The findings provide insights into the resonant behaviour of a CLAS and demonstrate how the mechanical and electrical properties of the dielectric substrate affect the CLAS’s performance. This research highlights the resilience of a CLAS to mechanical loadings, underscoring its potential for reliable long-term use in aerospace applications.

## 2. Materials and Methods

### 2.1. Specimen

**Figure 3:**
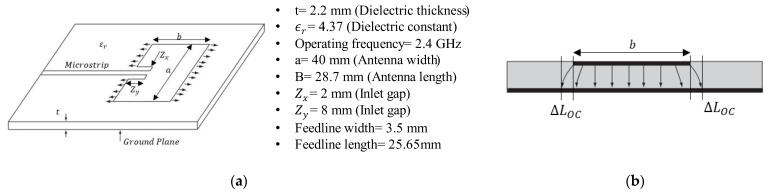
(**a**) Dimensions of microstrip antenna patch; (**b**) fringing field around the antenna [4].

Microstrip patch antennas were chosen for this investigation for their ease of design, manufacturing, and performance as a CLAS. We calculated the dimensions of a microstrip patch antenna based on the dielectric constant, dielectric height, and operating frequency, which were known to achieve a pre-determined resonant frequency. In this paper, the antenna shown in Figure 3a resonated at approximately 2.4 GHz, with a bandwidth of 22 MHz to support the IEEE 802.11 wireless communication protocol, as detailed in [15]. The bandwidth determines the upper and lower limits of the resonant frequency of the CLAS constructed with these parameters, which must not be exceeded when exposed to the mechanical loading used in this investigation.

The figure below illustrates the antenna and its dimensions. The inlet recesses (Zx and Zy) are crucial for impedance matching, thereby optimising the antenna’s performance. Figure 3b shows a non-uniform electric field distributed around the antenna, forming a fringing field that extends beyond the edges of the antenna structure, affecting the antenna’s overall radiation pattern and performance.

**Figure 4:**
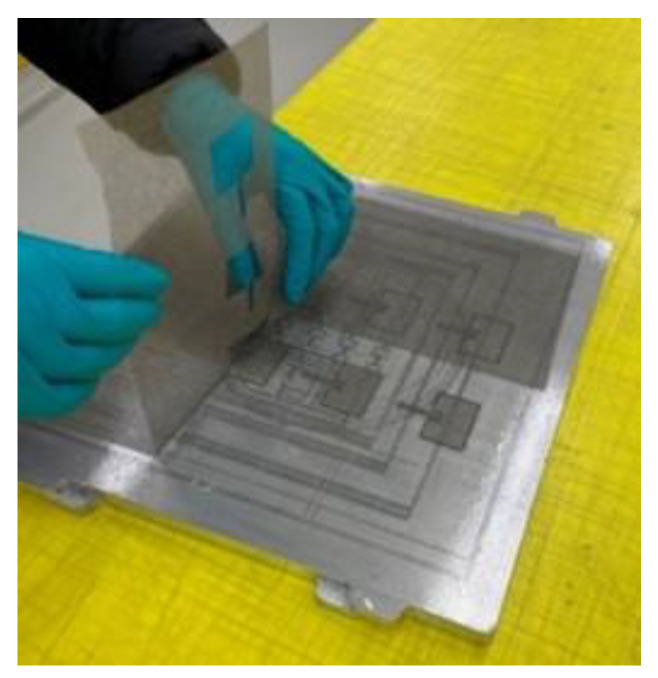
Laser-cut patch antennas prior to electroplating with copper.

A non-woven veil material offers a significant advantage over conventional copper foil in resistance to load and fatigue with less degradation in electromagnetic performance. The material used for the antenna patch in this paper is a non-woven carbon veil manufactured by TFP and copper-electroplated by the Australian Defence Science and Technology Group (DST Group). This material was also used to fabricate a CLAS in [16,17]. The veil material is lightweight and usually 0.15 mm thick. Antennas were precisely laser-cut to the dimensions using a Trotec SpeedMarker 700 (Trotec Laser GmbH, Marchtrenk, Austria). Figure 4 shows the parent material being removed from the antenna delicately.

A 2.2 mm thick laminate was selected as the substrate for the patch antenna for its dielectric properties. This substrate was fabricated using six layers of GMS EP-280 S-Glass glass fibre-reinforced polymer (GMS composites, Chadstone, VIC, Australia). Each cross-weave ply was 0.275 mm in thickness. The plies were cut to the dimensions of a 450 × 450 mm panel with ±45° fibre orientation using a Pathfinder L-series ply cutter. Once the composite was cured and consolidated, it was cut into multiple individual coupons with dimensions of 146 mm × 60 mm by CNC router machining with a burr cutter. These coupons were then prepared with antenna material in two specimen configurations, A and B, which are detailed below.

#### 2.1.1. Configuration A: Removeable Antenna Patch (RAP)

**Figure 5:**
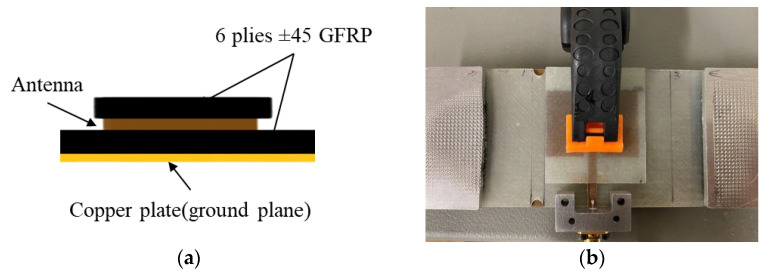
Configuration A: Removeable Antenna Patch: (**a**) schematic diagram; (**b**) experimental setup.

Figure 5a shows the antenna/specimen/copper-plate arrangement of Configuration A. In this configuration, an antenna patch was bonded to a small 6-layer GFRP substrate and pressed onto the target GFRP using an antenna clamp, as illustrated in Figure 5b. This clamping method provides a consistent antenna–substrate interface across tests. The antenna patch was positioned in the centre of the GFRP substrate, with the top edge 10 mm away from the substrate edge. We use this antenna patch arrangement to assess the effects of substrate fatigue on the resonant frequency behaviour. In this setup, only the substrate was subjected to loading. The antenna patch was applied to the specimen after each load excursion to investigate how the damage accumulated by the GFRP affects the resonant frequency response of the CLAS.

#### 2.1.2. Configuration B: Surface-Mounted Antenna Patch (SMAP)

**Figure 6:**
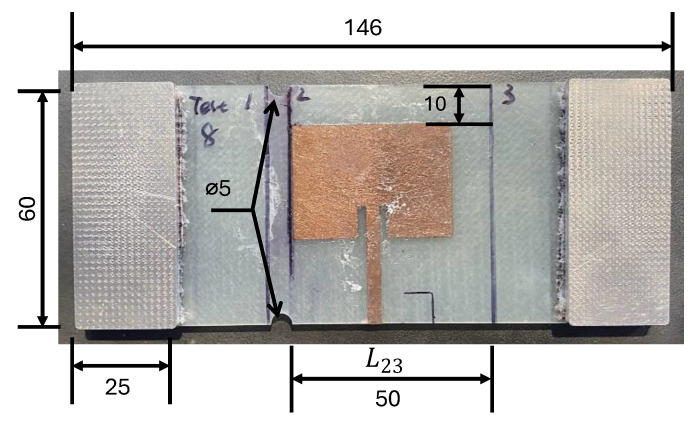
Configuration B: Surface-Mounted Antenna Patch (SMAP) with aluminium tabs at each end.

In this configuration, we placed and bonded the antenna on the GFRP substrate using cyanoacrylate at a pre-determined location similar to the RAP configuration to maintain consistency across experiments, illustrated in Figure 6. The antenna will experience mechanical loading with the substrate simulating an integrated antenna in this configuration, as demonstrated in [14].

#### 2.1.3. S11 Curves for Configurations A and B

The antenna input reflection coefficient (S11) of the specimens was measured to assess the resonant behaviour of the CLAS. A copper plate was used as the ground plane of the CLAS to eliminate errors induced by damage to the ground plane and to maintain consistency. The measurements were taken using a NanoVNA V2 Plus 4 (HCXQS group, Nanjing, China) with an SMA connector attached to the antenna feedline, as shown in Figure 5b.

**Figure 7:**
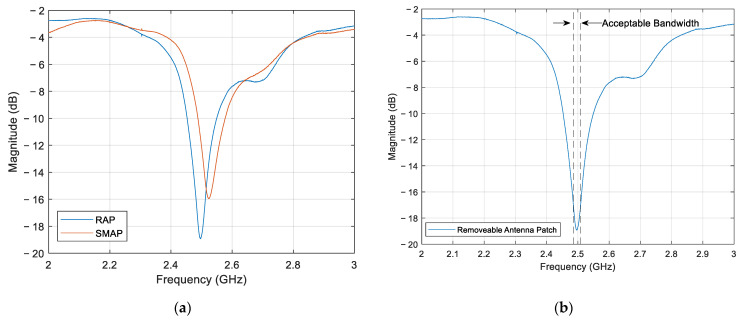
S11 curves for: (**a**) RAP and SMAP; (**b**) RAP and its acceptable bandwidth.

Figure 7a shows the S11 curves between the RAP and SMAP configurations. The RAP and SMAP configurations exhibited a resonant peak at 2.49 and 2.52 GHz, respectively. Figure 7b presents the S11 parameter for the RAP configuration, with the upper and lower frequency limits defined by the acceptable frequency bandwidth of 22 MHz.

### 2.2. Correlation between Resonant Frequency and Substrate Dielectric Properties

The fundamental equation of resonant frequency $f_{r}$ for a CLAS can be expressed as:

(1) $$f_{r}=\frac{c}{2\sqrt{\epsilon_{eff}}\left(b+2∆L_{OC}\right)}$$

where c is the speed of light in vacuum, a,b, and t are the dimensions of the CLAS (shown in Figure 3a), and $\epsilon_{eff}$ is the effective permittivity of the substrate, which is defined in Equation (2) [18]:

(2) $$\epsilon_{eff}=\frac{\epsilon_{r}+1}{2}+\frac{\epsilon_{r}-1}{2}{\left(1+\frac{10t}{a}\right)}^{-0.5}$$

where $\epsilon_{r}$ is the relative permittivity which is defined as the ratio of the material’s permittivity (ϵ) to the permittivity of free space ($\epsilon_{0}$) as shown below:

(3) $$\epsilon_{r}=\frac{\epsilon }{\epsilon_{0}}$$

The effective length of the fringing field as shown in Figure 3b is defined as:

(4) $$\frac{∆L_{OC}}{t}=0.412\frac{\left(\epsilon_{eff}+0.3\right)\left(\frac{a}{t}+0.264\right)}{\left(\epsilon_{eff}-0.258\right)\left(\frac{a}{t}+0.813\right)}$$

**Figure 8:**
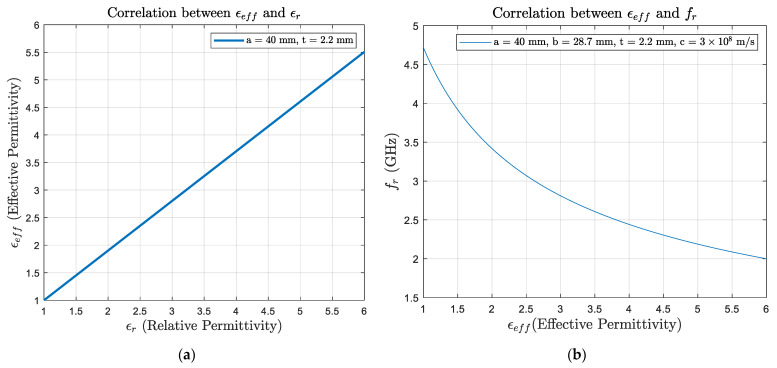
Correlations between (**a**) relative permittivity $\epsilon_{r}$ and effective permittivity $\epsilon_{eff}$, and (**b**) resonant frequency $\;f_{r}$ and substrate effective permittivity $\epsilon_{eff}$ under the condition of CLAS’s fixed geometry.

The equation above shows that resonant frequency depends on the antenna patch dimensions and the substrate electromagnetic properties. Supposing that there is no change in the geometry of the antenna and the thickness of the substrate, the correlation between the relative permittivity $\epsilon_{r}$ and effective permittivity $\epsilon_{eff}$ is close to linearity, which is shown in Figure 8a, where the $\epsilon_{r}$ value ranges from 1 (air) to 4.37 (the substrate used in this study: GFRP). In addition, the relationship between resonant frequency $\;f_{r}$ and substrate effective permittivity $\epsilon_{eff}$ is shown in Figure 8b. Therefore, changes in the electromagnetic properties of the substrate could significantly affect CLAS performance, potentially causing the antenna to stray out of the acceptable bandwidth. Understanding the fatigue response of the substrate with its permittivity would help estimate the service life of the CLAS.

### 2.3. The Effects of Damage Accumulation on the Effective Permittivity of GFRP

**Figure 9:**
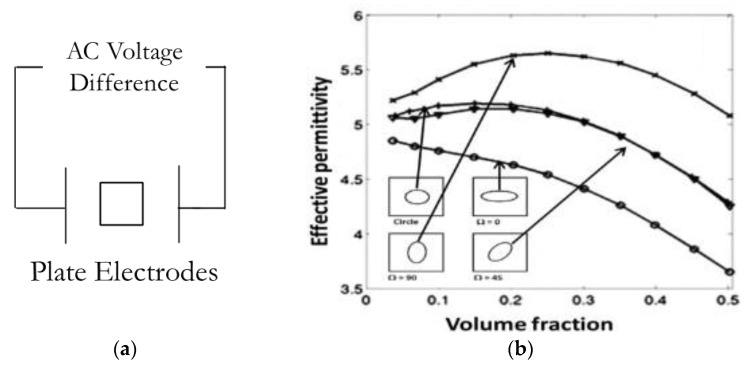
(**a**) Schematic diagram of parallel plate electrode simulation; (**b**) predicted variation of global effective permittivity with volume fraction of inclusions with lower permittivity than the matrix [19].

Regarding the GFRP substrate’s permittivity $\epsilon_{eff}$, variation due to damage accumulation is reported in the computational publications [19,20,21] and supported by experimental data in [22,23]. In the research [19] conducted by Raihan et al., they simulated a set of two fixed plate electrodes with AC voltage difference to calculate the capacitance of the composite material as shown in Figure 9a, consequently calculating the permittivity. As shown below (Figure 9b), an inclusion with relatively lower permittivity was placed inside the matrix to simulate the damage accumulation. In addition to the circular shape inclusion, they also considered inclusions with oval shapes and different angular orientations. The result demonstrated a correlation between the inclusion’s volume fraction and the composite’s effective permittivity. There are a few features to note in the plot. The oval shape inclusion with an orientation of 45-degree has a similar result to the circular particles. The permittivity slightly increases at a low volume fraction, then significantly decreases when the volume fraction approaches 0.5. Similar behaviour is also identified in the 90-degree condition, but the non-linear correlation is more pronounced at low-volume fractions. The 0-degree condition demonstrates a close to linear correlation between the volume fraction and effective permittivity. This simulation shows that as the size of inclusion increases (damage accumulates) in the composite, the effective permittivity does not necessarily increase monotonically, and the behaviour is highly dependent on the inclusion orientation. Similar results are also reported in their COMSOL simulation papers [20,21].

**Figure 10:**
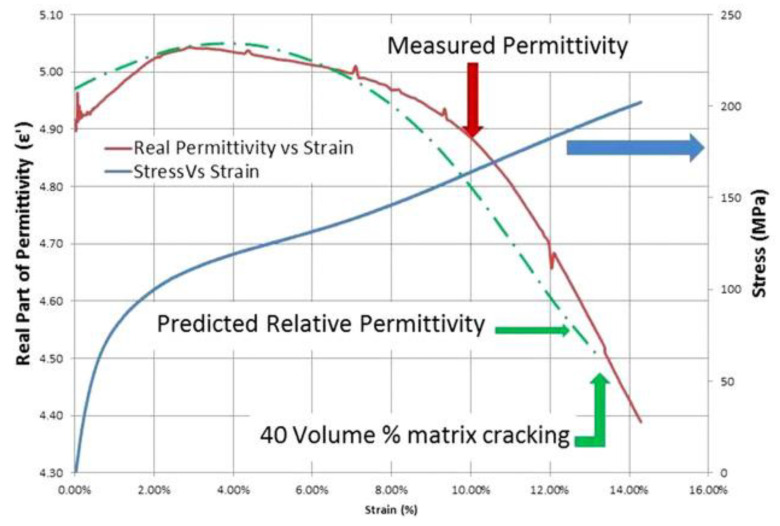
Response of the dielectric property in different zones of damage progression [22].

In another work by Raihan, R. et al. [22], they measured a ±45° GFRP specimen’s dielectric property while it underwent static tensile loading. The results, illustrated in Figure 10, show the correlation between specimen strain and relative permittivity during uniaxial tensile testing. Specifically, the results indicate that the permittivity increase occurs during the initial stages of the damage accumulation and is attributed to the matrix’s microcracking. The permittivity subsequently decreases when matrix cracking saturates, and fibre trellising commences, followed by a significant drop when fibre fracture starts, leading to specimen failure, with similar findings reported in [23].

### 2.4. Experimental Methods

This paper investigates how the reported response of the GFRP permittivity to damage accumulation affects a CLAS when subjected to mechanical loading. The investigation focuses on the effects of two mechanical loading conditions (quasi-static tension and cyclic fatigue) on a CLAS to understand the relationship between damage accumulated in the GFRP substrate and the resonant behaviour of the CLAS.

Early tensile tests failed at the grip section of the test specimens. To address this, two notches with a 5 mm diameter were created 2.5 mm away from the antenna at the top and bottom edges, as shown in Figure 5b and Figure 6, to induce damage at the location of interest near the patch antenna. Two pairs of aluminium tabs were attached to the specimen using a rough material to provide a gripping area for the tests. With these adjustments, a 6-ply GFRP specimen was loaded in the MTS 810 machine at a 0.5 mm/min displacement rate until failure occurred to obtain the force-displacement curve as guidance for subsequent mechanical tests. This force-displacement curve allows us to establish the loading steps for the quasi-static tests to reveal the CLAS’s response to progressive GFRP damage.

During the quasi-static tensile tests, the specimen was loaded to a pre-determined displacement level at a 0.5 mm/min rate. A slow loading rate will ensure evenly distributed damage across the specimen [24]. At each pre-set displacement step, the specimen was removed from the test machine to conduct an S11 measurement and to measure the length $L_{23}$ between lines 2 and 3, as marked in Figure 6, at the location of the antenna patch. The residual strain $\varepsilon_{residual}$, shown in Equation (5), is used to denote the extent of damage sustained at the antenna location.

(5) $$\varepsilon_{residual}=\frac{∆L}{L_{23}}$$

where $\varepsilon_{residual}$ is the residual strain at the location of antenna patch, $L_{23}$ is the original length between lines 2 and 3. ∆L is the change in $L_{23}$, measured after each step.

Following the S11 and length measurements, the specimen was reinstalled in the test machine and loaded to a higher pre-set displacement level to induce more damage (i.e., with a higher residual strain level, $\varepsilon_{residual}$). In this set of quasi-static tests, we tested three specimens of each of the two configurations of CLAS (i.e., Configuration A and Configuration B).

**Figure 11:**
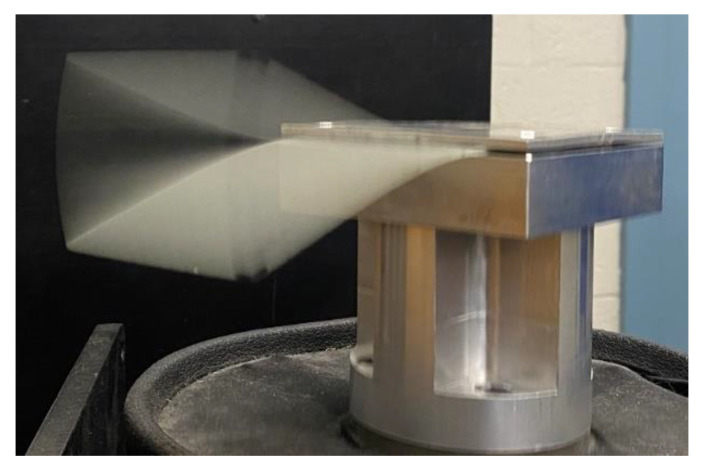
First mode vibration of GFRP induced by mechanical shaker.

A series of cyclic fatigue experiments were conducted to support the quasi-static test, where the test specimen was placed in an electromechanical shaker, as shown in Figure 11, and excited in its first natural mode of vibration. The vibration amplitude delivered a bending tensile strain of approximately 8000 µε at the fixed edge. In contrast to the damage induced by the quasi-static test that resulted in significant specimen elongation, the highly localised damage caused by this fatigue test arrangement was readily identified through visual inspection. The S11 measurements were conducted every 400k cycles in the fatigue experiment. Similar to the quasi-static tests, the cyclic fatigue experiments were performed with the two CLAS configurations.

## 3. Experimental Results

### 3.1. Tensile-to-Failure Test

**Figure 12:**
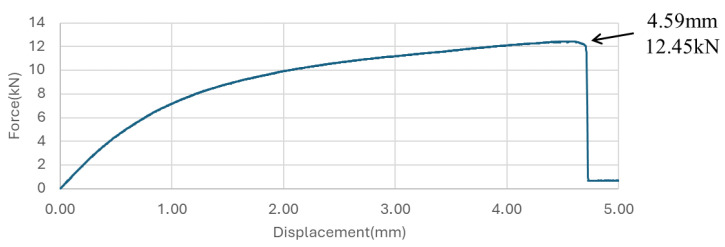
Force-displacement curve for ±45° GFRP specimen under tensile loading.

Figure 12 shows the results presenting the non-linear displacement-load behaviour of ±45° GFRP, indicating that the specimen failed at a force of 12.45 kN with a corresponding displacement of 4.59 mm. Initially, the relationship between force and displacement exhibits near-linearity up to a force of 6 kN. Beyond this point, significant non-linearity becomes evident, suggesting that the GFRP has exceeded its elastic limit and begun to accumulate damage. The onset of this non-linear behaviour marks the commencement of damage accumulation within the GFRP material. We used this load-displacement curve to help set the target displacement levels for quasi-static tests.

### 3.2. Quasi-Static Tensile Tests

**Figure 13:**
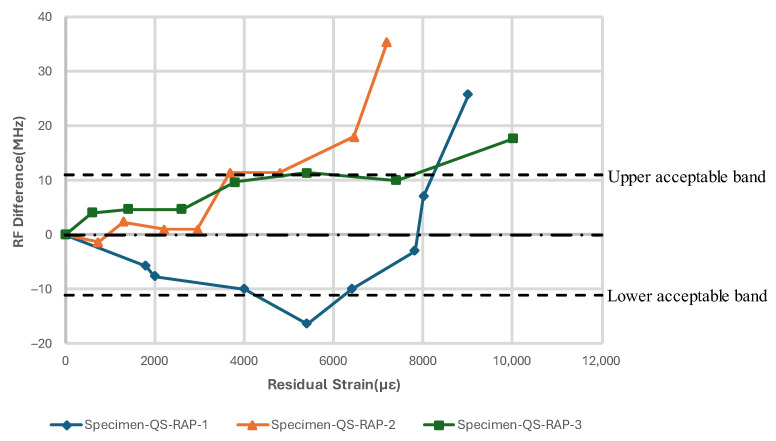
Resonant frequency shift of CLAS due to quasi-static uniaxial tensile loading with RAP.

**Figure 14:**
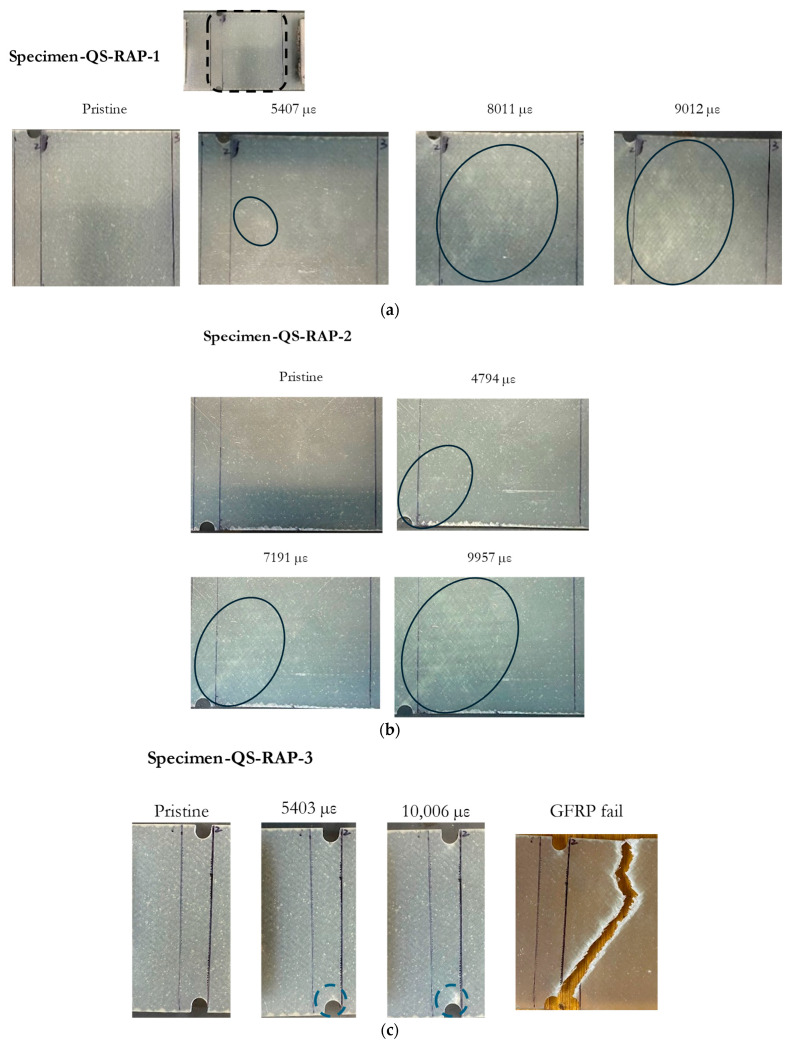
Damage accumulation on specimen across residual strain value for: (**a**) Specimen-QS-RAP-1; (**b**) Specimen-QS-RAP-2; (**c**) Specimen-QS-RAP-3.

**Figure 15:**
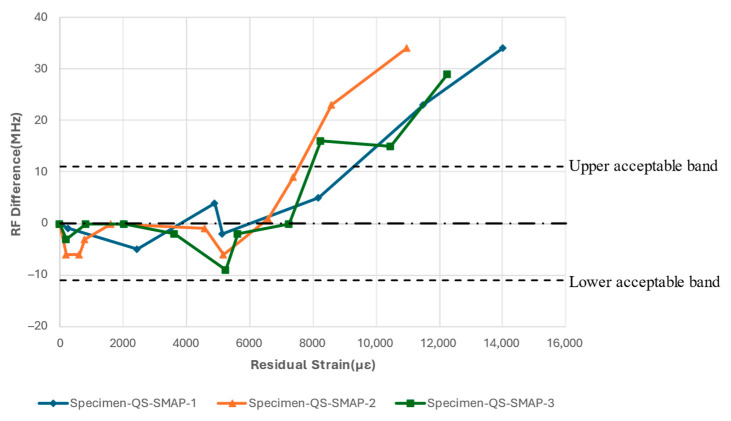
Resonant frequency shift of CLAS due to quasi-static uniaxial tensile loading with SMAP.

**Figure 16:**
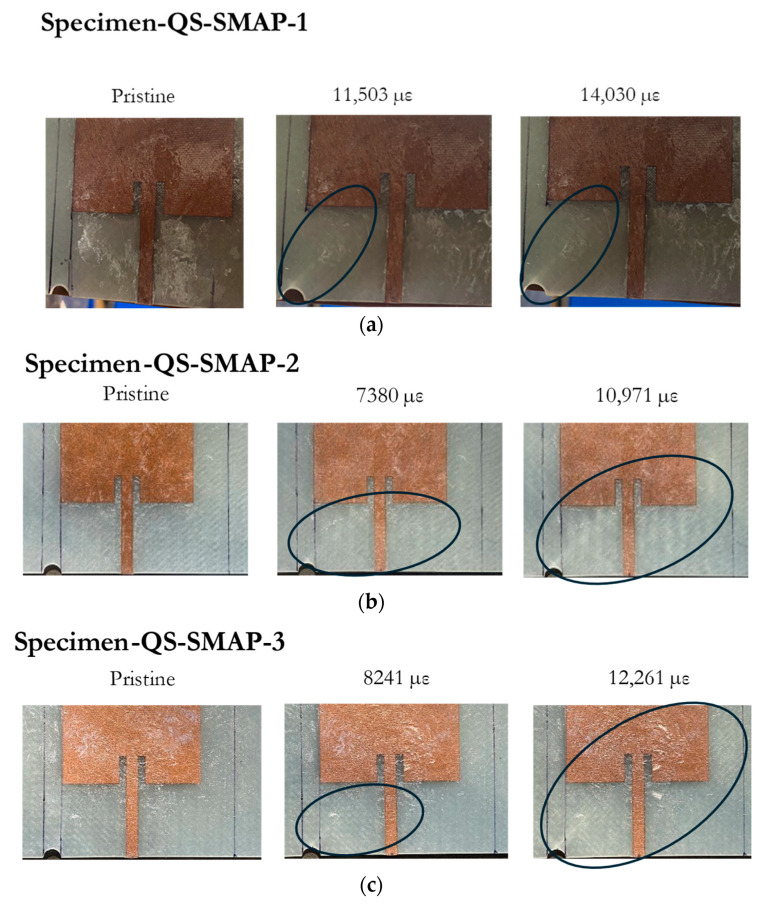
Damage accumulation on specimen across residual strain values for: (**a**) Specimen-QS-SMAP-1; (**b**) Specimen-QS-SMAP-2; (**c**) Specimen-QS-SMAP-3.

Figure 13, Figure 14, Figure 15 and Figure 16 show the resonant frequency response to the damage induced by the tensile load and damage accumulation on specimen for Configurations A and B, respectively. Generally, the resonant frequency decreases at the beginning of the test; then, when the residual strain, $\varepsilon_{residual}$, in the vicinity of the antenna, reaches between 4000 µε and 6000 µε, the resonant frequency starts to increase and exceeds the acceptable bandwidth at the end of the tests. The subsequent sections will present a detailed description of the results for each configuration specimen.

#### 3.2.1. Test Configuration A (Removeable Antenna Patch, RAP)

The resonant frequency measured with Specimen-QS-RAP-1 showed a decrease of 17 MHz from its centre frequency, when the residual strain reached approximately 5407 µε. This frequency shift coincided with minor damage observed beneath the antenna, as highlighted by ovals in Figure 14a. As the test progressed and the residual strain continued to increase at the antenna’s location, the whitened region of the GFRP became more pronounced. The resonant frequency of the specimen rose as the damage propagated across the specimen, eventually moving out of the band at $\varepsilon_{residual}\sim$8000 µε. The experiment concluded at 9012 µε without fracture.

Specimen-QS-RAP-2’s resonant frequency did not change significantly at low residual strain values. However, the specimen’s resonant frequency increased when the residual strain, $\varepsilon_{residual}$, reached approximately 4794 µε, which coincided with similar whitening around the antenna area, as shown in Figure 14b. As the residual strain increased, resonant frequency moved out of the bandwidth at $\varepsilon_{residual}=$ 6453 µε, and the experiment concluded at 9957 µε without GFRP failure.

The resonant frequency of Specimen-QS-RAP-3 rose with increasing residual strain on the GFRP and traversed out of the acceptable bandwidth at a residual strain of approximately 7400 µε. Unlike Specimen-QS-RAP-1 and -2, localized damage appeared at the notch area, eventually forming a crack, which is marked with a blue dashed circle. This crack led to the GFRP specimen fracturing at the end of the test, as shown in Figure 14c.

#### 3.2.2. Test Configuration B (Surface-Mounted Antenna Patch, SMAP)

The quasi-static tensile tests were repeated with an antenna patch bonded to the GFRP substrate. The tests with the antenna bonded to the GFRP demonstrated a similar trend of resonant frequency fluctuations at the beginning of the tests. Initially, the resonant frequency fluctuated between 0 and −5 MHz before the value of the residual strain, $\varepsilon_{residual}$, reached approximately 5000 µε, as shown in Figure 15. During this period, no apparent damage was observed around the notch on the substrate based on visual inspection. Beyond a residual strain, $\varepsilon_{residual}$, of 5000 µε, the resonant frequency of all three specimens increased and eventually strayed out of the band when the residual strain, $\varepsilon_{residual}$, exceeded 8000 µε. The images in Figure 16 indicate how damage accumulates on the substrate at the various stages of the test, as indicated by the residual strains; as the strain increases, the visual damage to the substrate becomes more pronounced, with a significant whitened region evident in the later stages of the tests, marked by the ovals.

Based on the resonant frequency relationship in Equation (1), the initial reduction in resonant frequency can be attributed to the increased effective permittivity of the GFRP during the early stages of damage and change in the length parameter, b. However, during this period, the change in b is insignificant. At the later stages of the tests, where the residual strain is significant, the reduction in both the effective permittivity and the length parameter will increase the resonant frequency of the patch antenna.

These tests indicate that the resonant frequency of a CLAS is influenced by the damage accumulated by the GFRP substrate, which affects its electrical properties in the manner shown in Figure 10. A significant finding is that both the SMAP and RAP test configurations show that the antenna’s resonant frequency strayed out-of-band at similar residual strain levels, suggesting that this behaviour is related to the substrate’s damage mechanism and cannot be attributed to the damage to the antenna material.

### 3.3. Cyclic Fatigue Experiments

The quasi-static tensile experiments described above resulted in significant specimen elongation during the tests. When used in aircraft structures, the antenna will be installed on surfaces that are unlikely to elongate to the same extent as in the tensile tests. However, the location of the antenna will still experience localised fatigue. This part of the investigation aims to study how the accumulation of localised fatigue will affect the resonant frequency of the patch antenna.

These tests were conducted with two types of CLAS specimens. In this series of tests, the specimens were not notched, as the damage was confined to the fixed region of the test specimens. Furthermore, the residual strain after each cyclic fatigue step was too small to be accurately recorded. This relatively small residual strain and the localized nature of the damage allowed us to observe how the resonant frequency responds as the damage gradually approaches the antenna location.

#### 3.3.1. Test Configuration A (Removeable Antenna Patch, RAP)

**Figure 17:**
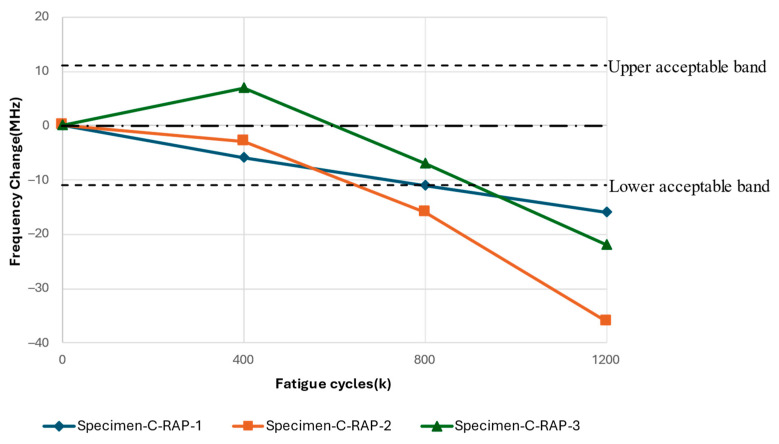
Resonant frequency shift of CLAS due to fatigue of substrate with RAP.

**Figure 18:**
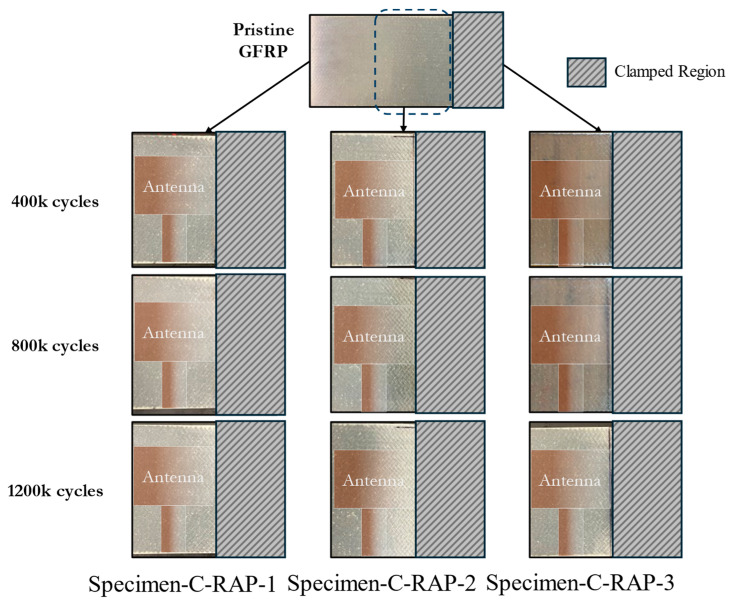
Damage accumulation on substrate for Specimen-C-RAP-1, -2, and -3.

The results in this section pertain to the fatigue experiments performed using a replacement antenna patch to study the effects of the fatigue of the GFRP on the resonant response of the CLAS system. The frequency difference relative to the fatigue cycles experienced by the substrate is reported in Figure 17. The antenna’s resonant frequency decreased with increasing fatigue cycles for all three tests. Figure 18 highlights the damage accumulation in the GFRP adjacent to the clamp region, marked with a dashed square in the pristine GFRP condition, and clearly indicates the antenna position on each specimen. The photos at 400k cycles can identify the whitened region’s initiation. Less damage was observed on Specimen-C-RAP-1 and Specimen-C-RAP-3, while Specimen-C-RAP-2 exhibited a larger whitened area around the fixed end of the GFRP compared to the other two specimens. The damage accumulation qualitatively correlates with the shift in resonant frequency, where Specimen-C-RAP-2 exhibited the most significant resonant frequency decrease of 35 MHz, with a notable whitening area occurring near the antenna. Specimen-C-RAP-1 showed only a 16 MHz change. Specimen-C-RAP-3 exhibited a resonant frequency decrease of 22 MHz, falling between the other two specimens, consistent with the observed damage accumulation. These results demonstrated that damage on the substrate, represented by the white pattern, reduced the resonant frequency of the CLAS. Using this antenna configuration, where the antenna patch remained pristine throughout the tests, the decrease in resonant frequency reflected the increase in the dielectric property of GFRP, according to Equation (1). The response of GFRP to the damage is consistent with the behaviour shown in Figure 10.

#### 3.3.2. Test Configuration B (Surface-Mounted Antenna Patch, SMAP)

**Figure 19:**
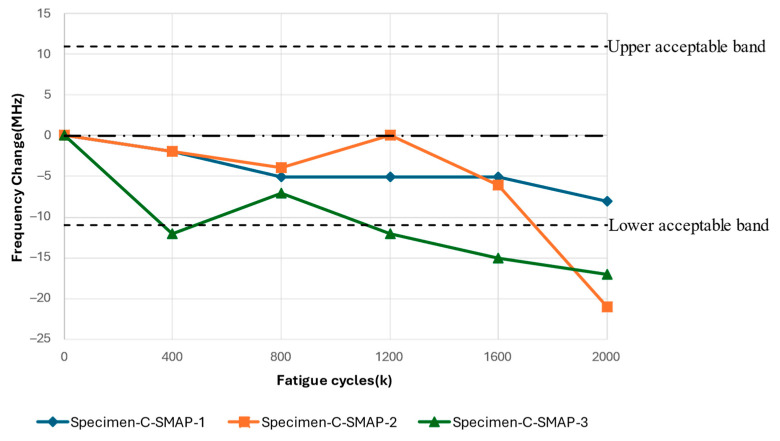
Resonant frequency shift of CLAS due to fatigue of substrate with SMAP.

The results in this section pertain to the fatigue experiments performed to study the response of the surface-mounted antenna patch to the accumulation of fatigue damage on the CLAS. The results of cyclic fatigue with an SMAP configuration, as shown in Figure 19, illustrated a similar frequency change as a function of fatigue cycles as the result with RAP. All specimens demonstrated a general reduction in the resonant frequency with increasing fatigue cycles. Specimen-C-SMAP-1 displays minor fluctuations, remaining close to −5 MHz for most fatigue cycles and ending near −8 MHz at 2000k cycles. Specimen-C-SMAP-2 demonstrated more variability, with slight fluctuations around −5 MHz up to 1200k cycles, followed by a more pronounced decline, reaching above −20 MHz by the end of the test. Specimen-C-SMAP-3 demonstrated a significant initial decrease after 400k cycles, then it dropped out of acceptable bandwidth after 1200k cycles. In the end, it decreased over 20 MHz at 2000k cycles.

**Figure 20:**
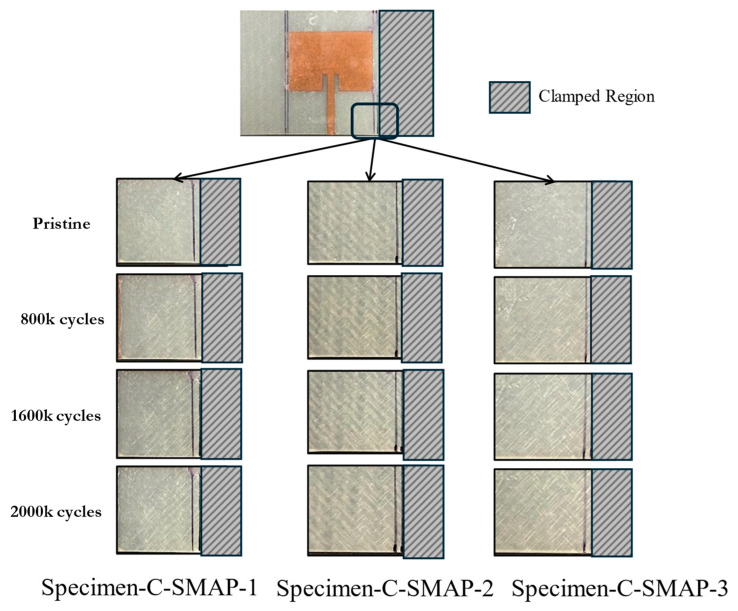
Damage accumulation on substrate for Specimen-C-SMAP-1, -2, and -3.

The difference between Specimen-C-SMAP-1, -2, and -3 correlates with the extent of damage on the GFRP substrate, shown in Figure 20. Based on visual inspection, Specimen-C-SMAP-2 exhibited more pronounced damage toward the right side where the antenna is located, indicated by significant whitening on the substrate, compared to Specimen-C-SMAP-1 and Specimen-C-SMAP-3. This increased damage in Specimen-C-SMAP-2 corresponded to a more substantial decrease in resonant frequency, as depicted in the previous plot. These results showed a reduction in the resonant frequency of the CLAS as the damage gradually progressed across the specimens. This further validates that damage to GFRP affects its permittivity, which is reflected in the change in the resonant frequency of the CLAS.

## 4. Discussion

In this study, GFRP is used to construct a CLAS system with non-woven antenna material. As discussed, the relative permittivity, $\epsilon_{r}$, of the substrate is one parameter that determines the CLAS’s resonant frequency. The literature reviewed above showed that the relative permittivity, $\epsilon_{r}$, of GFRP is affected by the damage it sustains. The results presented above show how the resonant frequency of the CLAS responds to the damage induced by mechanical loading on the substrate. In the investigation, decoupling the antenna from the substrate was essential to evaluate the integrated antenna system’s actual response, and these experiments were repeated with the antenna patch mounted permanently on the substrate.

In the quasi-static tests with the RAP configuration, which decoupled the antenna from the CLAS and kept the antenna under pristine conditions throughout the tests, the isolation of the antenna patch in this configuration reduced the likelihood of mechanical loading affecting the antenna patch, a conclusion previously demonstrated by Healey [4].

Three specimens underwent identical testing procedures to ensure reproducibility. Despite the uniform testing conditions, notable differences emerged in how damage accumulated on the specimens at low residual strain levels. This was evidenced by the initiation of whitened regions, which led to variations in the resonant frequency response to the damage.

For Specimen-QS-RAP-1, damage initiated at the centre of the specimen beneath the antenna at low strain values, coinciding with a decrease in resonant frequency. In the case of Specimen-QS-RAP-2, damage originated at the notch, accompanied by fluctuations in frequency. As the test progressed and the residual strain increased, the whitened region propagated across the specimen for both Specimen-QS-RAP-1 and -2. Concurrently, the resonant frequency began to rise and eventually exceeded the acceptable bandwidth.

Specimen-QS-RAP-3 exhibited damage initiation at the notch similar to Specimen-QS-RAP-2. However, a crack developed at the notch, indicating a difference in the damage mechanism compared to Specimen-QS-RAP-1 and -2. This difference was also revealed in the monotonically increasing resonant frequency observed until the specimen failed at the end of the test.

The differences in the behaviour of resonant frequency between specimens reveal that damage to GFRP affects its dielectric properties, thereby influencing the resonant frequency of the CLAS. It also demonstrates that different damage mechanisms affect the resonant frequency response to mechanically induced damage.

The damage accumulation pattern and resonant frequency response to the quasi-static loading of the SMAP specimen configuration showed a similar trending to that observed in the Specimen-QS-RAP-2. In this case, the damage initiated at the notch region at low strain values, coupled with fluctuations in the resonant frequency. As the residual strain, $\varepsilon_{residual}$, sustained by the GFRP increased further, and exceeded 6000 µε, the reduction in the relative permittivity, $\epsilon_{r}$, led to an increase in resonant frequency according to Equation (1). It is important to note that the residual strain level that caused the resonant frequency of both antenna configurations to stray out of the band is similar, indicating that the damage induced on the substrate is the primary cause.

The significant elongation of the specimens in quasi-static experiments is unlikely to occur at the location when the antenna is integrated into aircraft structures. However, localised fatigue due to operational loads could affect the performance of GFRP. To address this, we conducted cyclic fatigue experiments using the electromagnetic shaker, which induced highly localised damage in the region adjacent to the antenna’s edge. Results from both RAP and SMAP configurations indicated that, under relatively small residual strains experienced by the specimens, the resonant frequency decreased as whitening damage on the GFRP progressively built up. This behaviour correlates with the observed increase in permittivity at low strain levels (Zone II), as depicted in Figure 10. The consistent observation between RAP and SMAP configurations demonstrated that the increase in the permittivity of the GFRP due to damage progression is the primary factor contributing to the decrease in resonant frequency.

Combining the results observed from the quasi-static tensile tests and cyclic fatigue experiments confirmed the existence of non-linear behaviour in the resonant frequency of the CLAS. The initial decrease in resonant frequency correlated with the accumulated damage on the substrate, indicating an increase in permittivity of GFRP. Following this initial stage, as further increases in residual strain occurred in the vicinity of the antenna patch, the resonant frequency increased, reflecting the reduction trend in the permittivity of the substrate. This response of permittivity to damage in GFRP agrees with the literature, evidencing the non-linearity in the permittivity of GFRP when subjected to mechanical damage.

These findings reveal the relationship between the damage process on the substrate and the resonant frequency of a CLAS and aid in understanding the effect of quasi-static tension and cyclic fatigue loading on CLAS performance. The above results will set the scene and assist with interpreting future experimental results. This understanding is essential for predicting these structures’ service life and reliability, thereby contributing to the advancement of CLAS technology and its practical applications.

## 5. Conclusions

The study investigated the response of a CLAS to mechanical loading, focusing on the correlation between damage and shift in resonant frequency. By testing two CLAS configurations under quasi-static and cyclic fatigue conditions, the research confirmed the non-linear relationship between damage accumulation in GFRP and its dielectric properties, impacting the resonant frequency of the CLAS. The RAP configuration demonstrated that isolating the antenna from mechanical loading revealed the primary influence of substrate damage on resonant frequency. Both RAP and SMAP configurations in cyclic fatigue tests showed a clear trend where increased damage led to higher permittivity and decreased resonant frequency, aligning with the existing literature.

These findings enhance our understanding of the relationship between damage in the substrate and resonant frequency in a CLAS. They highlight the impact of mechanical loading on the substrate’s dielectric property, affecting the resonant frequency. The results are crucial for predicting a CLAS’s reliability and service life, aiding in the design and placement of patch antennas on load-bearing deformable surfaces. This work lays the groundwork for future experimental studies and advances the practical applications of CLAS technology.

## Data Availability

The datasets presented in this article are not readily available because the data are part of ongoing research. Requests to access the datasets should be directed to wing.kong.chiu@monash.edu.

## References

[1] You C., Hwang W., Park H., Lee R., Park W. (2003). Microstrip antenna for SAR application with composite sandwich construction: Surface-antenna-structure demonstration. J. Compos. Mater..

[2] Callus P.J. (2007). Conformal Load-Bearing Antenna Structure for Australian Defence Force Aircraft.

[3] Lockyer A.J., Alt K.H., Kudva J.N., Kinslow R.W., Goetz A.C. (1997). Conformal Load-Bearing Antenna Structures (CLAS): Initiative for Multiple Military and Commercial Applications. Smart Structures and Materials 1997: Smart Electronics and MEMS.

[4] Healey R., Nicholson K.J., Wang J., Patniotis J., Lynch T. (2022). Conformal Load-Bearing Antenna Structures—Mechanical Loading Considerations. Sensors.

[5] Nicholson K., Dunbabin O., Baum T., Ghorbani K. (2017). Characterisation of integrated microstrip lines in aerospace composite structure. Electron. Lett..

[6] Baum T.C., Ziolkowski R.W., Ghorbani K., Nicholson K.J. Investigation of a conformal amplifier embedded in an aerospace composite structure. Proceedings of the 2017 47th European Microwave Conference (EuMC).

[7] Mehdipour A., Sebak A.R., Trueman C.W., Rosca I.D., Hoa S.V. Conductive carbon fiber composite materials for antenna and microwave applications. Proceedings of the 2012 29th National Radio Science Conference (NRSC).

[8] Rudd M., Baum T.C., Mapleback B., Ghorbani K., Nicholson K.J. (2017). Reducing the attenuation in CFRP waveguide using carbon fiber veil. IEEE Microw. Wirel. Compon. Lett..

[9] Todd A., Baum T., Nicholson K., Ziolkowski R., Ghorbani K. Towards Carbon Based Artificial Impedance Surfaces for Conformal Aerospace Applications. Proceedings of the 2018 48th European Microwave Conference (EuMC).

[10] Kim H., Park M., Hsieh K. (2006). Fatigue fracture of embedded copper conductors in multifunctional composite structures. Compos. Sci. Technol..

[11] Kim H., Hsieh K. (2012). Measurement and prediction of embedded copper foil fatigue crack growth in multifunctional composite structure. Compos. Part A Appl. Sci. Manuf..

[12] Beziuk G., Krajewski A., Baum T.C., Nicholson K.J., Ghorbani K. (2023). Electromagnetic and Electronic Aerospace Conformal Load-Bearing Smart Skins: A Review. IEEE J. Microw..

[13] You C.S., Hwang W. (2005). Design of load-bearing antenna structures by embedding technology of microstrip antenna in composite sandwich structure. Compos. Struct..

[14] Lu S., Nicholson K., Patniotis J., Wang J., Kong W. (2023). Fatigue response of conformal load bearing antenna structures. Mater. Res. Proc..

[15] Kim J., Jeon W., Park K.-J., Choi J.P. (2018). Coexistence of full-duplex-based IEEE 802.15. 4 and IEEE 802.11. IEEE Trans. Ind. Inform..

[16] Baum T.C., Ziolkowski R.W., Ghorbani K., Nicholson K.J. (2017). Investigations of a load-bearing composite electrically small Egyptian axe dipole antenna. IEEE Trans. Antennas Propag..

[17] Baum T.C., Nicholson K.J., Ghorbani K., Ziolkowski R.W. Passive and active metamaterial-inspired radiating and scattering systems integrated into structural composite materials. Proceedings of the 2017 11th International Congress on Engineered Materials Platforms for Novel Wave Phenomena (Metamaterials).

[18] Balanis C. (2016). Antenna Theory: Analysis and Design.

[19] Raihan R., Reifsnider K., Cacuci D., Liu Q. (2015). Dielectric signatures and interpretive analysis for changes of state in composite materials. ZAMM-J. Appl. Math. Mech. Z. Für Angew. Math. Mech..

[20] Raihan R., Rabbi F., Vadlamudi V., Reifsnider K. (2015). Composite materials damage modeling based on dielectric properties. Mater. Sci. Appl..

[21] Vadlamudi V., Raihan R., Reifsnider K. (2017). Multiphysics based simulation of damage progression in composites. Mater. Sci. Appl..

[22] Raihan R., Adkins J.-M., Baker J., Rabbi F., Reifsnider K. (2014). Relationship of dielectric property change to composite material state degradation. Compos. Sci. Technol..

[23] Vadlamudi V., Shaik R., Raihan R., Reifsnider K., Iarve E. (2019). Identification of current material state in composites using a dielectric state variable. Compos. Part A Appl. Sci. Manuf..

[24] Fitoussi J., Meraghni F., Jendli Z., Hug G., Baptiste D. (2005). Experimental methodology for high strain-rates tensile behaviour analysis of polymer matrix composites. Compos. Sci. Technol..

